# A Holistic Review of Cyber–Physical–Social Systems: New Directions and Opportunities

**DOI:** 10.3390/s23177391

**Published:** 2023-08-24

**Authors:** Theresa Sobb, Benjamin Turnbull, Nour Moustafa

**Affiliations:** School of Systems and Computing, University of New South Wales, Canberra 2612, Australia; t.sobb@adfa.edu.au (T.S.); nour.moustafa@unsw.edu.au (N.M.)

**Keywords:** cyber–physical–social systems (CPSS), cyber–physical systems (CPS), cyber security, internet of things (IoT), social media, influence

## Abstract

A Cyber–Physical–Social System (CPSS) is an evolving subset of Cyber–Physical Systems (CPS), which involve the interlinking of the cyber, physical, and social domains within a system-of-systems mindset. CPSS is in a growing state, which combines secure digital technologies with physical systems (e.g., sensors and actuators) and incorporates social aspects (e.g., human interactions and behaviors, and societal norms) to facilitate automated and secure services to end-users and organisations. This paper reviews the field of CPSS, especially in the scope of complexity theory and cyber security to determine its impact on CPS and social media’s influence activities. The significance of CPSS lies in its potential to provide solutions to complex societal problems that are difficult to address through traditional approaches. With the integration of physical, social, and cyber components, CPSS can realize the full potential of IoT, big data analytics, and machine learning, leading to increased efficiency, improved sustainability and better decision making. CPSS presents exciting opportunities for innovation and advancement in multiple domains, improving the quality of life for people around the world. Research challenges to CPSS include the integration of hard and soft system components within all three domains, in addition to sociological metrics, data security, processing optimization and ethical implications. The findings of this paper note key research trends in the fields of CPSS, and recent novel contributions, followed by identified research gaps and future work.

## 1. Introduction

Cyber–Physical Systems (CPS) are increasingly becoming integration points for computation and are a significant part of people’s daily lives in their homes, businesses, governments, and industry. CPS were developed in order to address modeling challenges for control systems and embedded computing, focusing on efficiency optimization and secure components [1]. Not only have CPS come to represent industrial control systems, but a wide variety of systems at the interface of the digital and physical worlds, including medical devices, education devices, Internet of Things (IoT), smart cities, and transportation systems, among others [2,3,4]. A CPS integrates the computational and physical capabilities of systems, enabling those embedded systems to interact with sensors and actuators that exist in the physical world [4,5]. A CPS is characterized by its ability to interact with the physical world through a combination of control and computation, enabled through communication [5] (p. 1) and [1]. The term is broad, spanning several generations of technological growth and application development, and has evolved with technologies such as the Internet of Things (IoT).

CPS have traditionally been focused on systems that involve purely technical and physical components, with humans existing as external entities to the system, considering cyber aspects, including confidentiality, integrity and availability [6]. A CPS architecture is closely aligned with IoT, consisting of three layers: the perception layer, the network layer, and the application layer [4,6,7]. The perception layer is concerned with sensors and actuators that measure and collect data. The network layer is responsible for the communication of data between agents. Finally, the application layer is concerned with the processing of information and decision making. As the field of CPS grows and system boundaries extend beyond traditional Cyber–Physical components, there is the potential need for new architectural paradigms to be considered, especially including a security-by-design layer to be embedded in order to safeguard these layers efficiently.

With the prevalence of social media, a Cyber–Physical–Social System (CPSS) has emerged, concerned with identifying and understanding how humans integrate within systems that encompass the cyber, physical and social space [6,8]. Such systems extend beyond human-in-the-loop systems, where people interact with the system in switch-case-like predefined actions or decision support scenarios [9]. Instead, CPSSs include an understanding of the social ramifications and their feedback loops within the scope of the system boundary [6]. CPSSs are designed to offer seamless communication, coordination, and integration between the cyber, physical, and social elements, resulting in more effective system performance. This integration allows systems to adapt and respond to changing conditions, optimise resource allocation, and improve overall system performance and user experiences. A CPSS was defined as a Cyber–Physical System that exhibits the coordination, conjoining and integration of both human and social characteristics within a larger CPS [10] (p. 85). This further highlighted the complexity of CPSS, being exacerbated by the social system’s inclusion in the wider system boundary. This definition would be linked with three hypotheses, where the physical, mental, and artificial worlds combine, and highlighted the internet as a vector for this integration [10,11]. Pervasive Intelligent Spaces (PIS) would be also added in the definition to enable the interaction of all agents in real time and heavily rely on pervasive IoT technologies later expanded [10] (p. 86). This research highlights that social media serves as the new revolution for CPSSs, changing the way that societies and industries may function [10]. Each of the domains incorporated into CPSSs—social, physical, and cyber—individually meet the definition of complex systems. The large-scale and significant numbers of interactions between each of these domains create a strong system-of-systems dynamic [12]. Considering CPSS approaches therefore requires the use of a system-of-systems mindset, and will continue to do so in the future [13].

The field of CPSS attempts to bridge the gap between traditional CPS research and social complexity research. As CPS become more integrated into society within a system-of-systems mindset, social factors have the potential to effect the functioning of Cyber–Physical components in unpredictable ways. CPSS provides researchers with the opportunity to solve complex societal problems that are difficult to address using traditional methods. These include attempts to understand and model human behavior in online social media environments in addition to modeling systems that include real-time human feedback such as seen in smart cities and smart transportation [14,15].

Some of the emerging trends in CPSS include the development of smart cities, intelligent transportation systems, and smart healthcare systems [16,17,18,19]. The significance of CPSS lies in its potential to provide solutions to complex societal problems that are difficult to address through traditional approaches. CPSS can be used in numerous domains such as healthcare, transportation, energy conservation, disaster management, and environmental monitoring [17,20,21,22]. With the integration of physical, social, and cyber systems, CPSS can realize the full potential of IoT, big data analytics, and machine learning, leading to increased efficiency, improved sustainability and better decision making. Therefore, CPSS presents exciting opportunities for innovation and advancement in multiple domains, improving the quality of life for people around the world. In a CPSS, the cyber component involves the seamless integration of computational algorithms, communication networks, and software applications. The physical component includes physical infrastructure, devices, and sensors that collect and provide data to the system. The social component encompasses human interactions, preferences, and behaviors, which influence the system’s operation and outcomes

In a CPSS, sensors and actuators in the physical domain are often underpinned by IoT technologies. Security challenges posed by IoT-enabling technologies include signal integrity and accurate event actuation [23]. Thapa et al. addresses concerns related to these fundamental units in a complex CPS context, proposing security solutions for autonomous vehicle case studies to enable secure design [24]. However, the perception layer of a CPSS has the potential to exist conceptually beyond the physical domain, and within the cyber and/or social ones. For example, a human agent within a social network acts as a sensor to its environment, which may then inform feedback loops into the wider CPSS [25].

A CPSS represents a field that encompasses a complex system of systems. Social factors have the potential to create emergent behaviors that can induce instability into critical CPSS networks within the world. CPSS effects exist beyond the boundaries of traditional CPS. This can be exemplified with two case examples—Non-Fungible Tokens (NFTs) and the historical events of the Arab Spring. These case studies highlight the importance of CPSSs and the need to understand their interactions within a complex system-of-systems due to their potential wide-scale consequences across multiple domains.

The economic value of Non-Fungible Tokens (NFTs) is derived through social factors. They exist within the cyber domain, but have real physical-world consequences including economic and market impacts. The value of NFTs is created based on social perception of the cost/benefit trade-off of that token, and is heavily influenced by aesthetic human psychological values [26]. Of course, NFTs and similar digital ledger technologies exist within the cyber domain, but they also have physical-world impacts through economic returns and potential changes to the labor market [27,28,29]. Thus, the existence and financial impacts of NFTs demonstrate the consequences of CPSSs on the large scale system-of-systems that effects large-scale world dynamics.

The Arab Spring [30] also highlighted how CPSSs can induce emergent behaviors that have multi-domain impacts. Protests that initially began in Tunisia spread to other countries including Libya, Egypt, Yemen, Syria, and Bahrain. This information spread was fueled through social media platforms including Facebook and Twitter, enabling real-time dissemination of information across national boundaries and organization and mobilization of protests based on shared ideologies. During the Arab Spring, features of complexity were exemplified through sensitivity to initial conditions with the initial protest suicide of Tunisian Mohamed Bouazizi, the self-organization of political groups via the internet, adaptive interaction of protests as events evolved, and emergent behaviors including the coup of the Egyptian President Mohamed Morsi [31,32,33,34,35,36]. The three CPSS domains were also clearly present throughout the Arab Spring. The cyber component was a key enabler to the dissemination of information between protesters via social media. The social component manifested through the sharing of ideologies and political engagement by both citizens and governments. Finally, the physical domain manifested through physical protests, the Egyptian coup, the Tremeseh massacre, the Syrian civil war, and diverse government reactions and reform.

A CPSS poses several challenges from a scientific perspective. One of the main challenges is the integration of various components of the CPS within the CPSS context. These components include sensors, network components, and actuators [37]. The nature of CPSS components further requires that resource synchronization and optimization occur for performance—a complex task [38,39]. The development of secure and reliable communication protocols that can safely transmit data between different components is an issue [4,40]. The new trends in CPSSs are characterized by the use of advanced technologies and methods such as artificial intelligence, machine learning, blockchain, and IoT [41,42,43]. These technologies allow CPSSs to interact with their environment more intelligently, accurately, and efficiently. As a result, CPSSs can improve the performance of physical and social systems, enhance decision-making capabilities, and unlock new applications.

CPSSs face a variety of cyber security challenges. Some challenges are common with those underlying the IoT, such as node capture, routing attacks and data theft [44]. Because CPSSs include the social landscape, this provides greater opportunity for social engineering attacks that exploit human vulnerabilities, using tactics such as deception, manipulation, or influence [45,46,47]. CPSSs present unique cyber security challenges, where threats may arise through any of the cyber, physical or social agents within the system. Subsequently, conscious development of CPSSs within a secure digital technology mindset is essential to assuring system operation in the face of cyber threats [48]. Ensuring the security and privacy of CPSSs is crucial because of the high volume of sensitive data being processed and shared [49]. Protecting against cyber attacks, asserting data integrity, and maintaining user privacy are significant challenges. CPSSs include various interconnected components, such as sensors, devices, and networks [50]. Ensuring seamless interoperability between these components is a challenge because of differences in protocols, standards, and technologies. CPSSs would also need to handle large amounts of data, users, and interconnected systems, ensuring scalability to accommodate such growth while maintaining system performance and efficiency, which is a problem. A CPSS should be resilient to failures, disruptions, and adverse events [51]. Ensuring robustness and fault tolerance to maintain system functionality and minimize potential damages is a significant challenge [52]. CPSSs raise ethical concerns, such as the potential amplification of social biases, discriminatory practices, and the misuse of personal data. Addressing these challenges and promoting responsible and inclusive CPSS design and usage is essential, which we attempt to highlight in this study.

The ongoing advancements in CPSSs are expected to have a significant impact on various industries and aspects of daily lives [52]. As a consequence, understanding the scope of current CPSS research within these topics is essential for the development of future work and innovation in the research field. This is enhanced by CPSS’s potential for impact in diverse and interconnected domains, ranging from politics to economics, infrastructure and algorithm design [18,51,53]. This paper aims to holistically review CPSSs from three angles—cyber, physical and social—in order to determine their impact on system performance and how they offer secure and automated services to end-users and organisations in the era of artificial intelligence (AI). We define a CPSS as a system that integrates cyber, physical, and social elements to interact and collaborate with each other.

The key contributions of this work are structured as follows:

- We determine a CPS and its interaction with social media and its components, and to what level it influences human behaviors.
- We examine the distribution and intersection of research associated with CPSS, complex systems, social media, influence and cyber security applications.
- We discuss the recent advancements of CPSSs and how they enhance human activities and system performances.
- We describe the recent challenges and lessons learned and future research directions of CPSSs.

This paper discusses Cyber–Physical–Social Systems within the larger context of Cyber–Physical Systems and the impact of social complexity on these systems in Section 2. In Section 3, we discuss the related studies mixed between CPS, social media, and CPSS. The recent technologies with CPSS are explained in Section 4. This is followed by examining the most influential terms in Section 5. Section 6 describes the role of CPSS with recent smart applications. Research challenges and lessons learned are explained in Section 7. Finally, we conclude the paper in Section 8.

## 2. Cyber Physical Social System (CPSS)

We define a CPSS in a similar way to Wang [10], but in addition, we include security-by-design to each component as an extension of a Cyber–Physical-System that includes human social systems within its holistic system boundary. A CPSS, therefore, includes social and cognitive functions not considered within traditional CPS [54]. We define a CPSS as a systematic architecture that connects social features with human and network elements, with social attributes disseminating in accordance with node relationships, considering a secure design to the elements and fractures. In this way, the social space exists beyond just people, but also within their interactions, networks, and interactions.

We do not consider any of the competing definitions of CPSS that do not model social systems within their system boundary, and instead note that these systems interface with the CPS, as seen in the work of Zhou and Lin [55]. Competing definitions include considering CPSS as battery-supplied low-energy devices [55] or systems that highly conjoin communication, sensing, computing, and C2 (Command and Control) within larger human societal contexts [56]. The application of CPSSs spans various domains, including smart cities, transportation systems, healthcare, manufacturing, and entertainment. By combining cyber, physical, and social elements, CPSSs can enable advancements such as autonomous vehicles, smart grid systems, personalized healthcare, and immersive virtual environments. Overall, CPSSs represent an interdependent and interconnected system that leverages digital technologies, physical infrastructure, and social dynamics to create intelligent and adaptive systems capable of addressing complex societal challenges.

A further distinction should be made between the concept of CPSS and Cyber–Physical–Human Systems (CPHS). CPHS include humans in the loop and focus on the interaction between people and Cyber–Physical Systems [9]. CPHS play a crucial role in various scientific fields, including robotics, artificial intelligence, and human–computer interaction. They enable the development of advanced technologies such as autonomous vehicles, wearable devices, smart homes, and interactive virtual reality platforms. By leveraging CPHS, researchers aim to enhance human productivity, safety, and well-being in various domains such as healthcare, transportation, manufacturing, and entertainment. For example, in healthcare, CPHS can be used to monitor patients’ vital signs remotely, enabling early detection of health issues and prompt intervention. In transportation, CPHS can facilitate the development of self-driving cars that optimize traffic flow and improve road safety. However, CPHS rely on people who feature cognition, predictability and motivation without considering complex social factors [9]. CPHS are suited for applications where the human element of the CPS behaves in a defined way using defined options, and has been used heavily in modelling human-in-the-loop systems such as those at the National Aeronautics and Space Administration [9].

### 2.1. Cyber–Physical System

**Figure 1:**
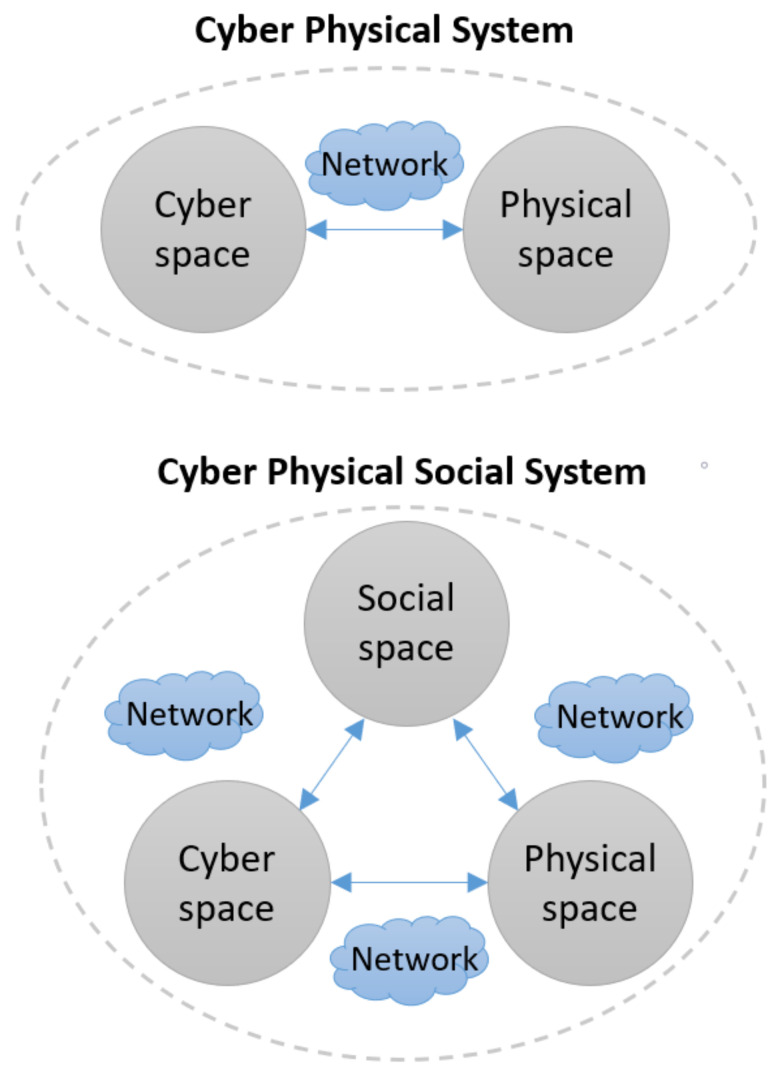
Evolution of the CPSS.

The ontology of CPSSs is difficult to define for two reasons. The first is that the boundaries between these agents or sub-systems are unclear. The second reason is that a CPSS includes human social systems within its holistic system boundary, as shown in Figure 1. Subsequently, modelling and measuring people’s social feelings and attitudes is more difficult than placing sensors in traditional Cyber–Physical Systems, because these sensors may need to exist at the interface or within the human mind itself [57]. The collection of data from social sensors is therefore a challenge within this field, as accurate metrics regarding social or emotional human states pose obstacles in terms of collection accuracy. In a traditional CPS, sensors can be implemented technically through technology paradigms such as IoT.

The connection between CPS IoT lies in their shared objective of connecting and exchanging data between physical devices. CPS involves the integration of sensors, actuators, and control systems, while IoT extends this concept by connecting a vast array of everyday objects or “things” through the internet. IoT acts as the backbone for CPS, providing the network infrastructure and communication protocols necessary to connect and exchange data between CPS components. This allows remote monitoring and control of physical processes in real time, enabling more efficient and autonomous decision making.

Converting human interactions and and behaviors in the social space into calculations inputted to sensors is a challenge within CPSS. Intrinsic social calculations that occur within the human mind are difficult to effectively record and calculate, and thus developing mechanisms to appropriately measure these social metrics is a field of continued CPSS research [57].

The combination of CPS with IoT leads to numerous applications across various domains, such as smart cities, transportation systems, healthcare, manufacturing, and energy management. By leveraging the capabilities of CPS and IoT, organizations can achieve improved efficiency, increased productivity, enhanced safety, and better resource management. The introduction of humans and their associated social contexts presents a unique challenge for CPSS research, as sensors to monitor these conditions need to quantify social data points [57]. An example of a CPS in this context would be a modern manufacturing facility, which would have computational sensors, actuators, and humans that would be making predefined or limited decisions based on the system state. By contrast, an example of a CPSS would be a humanitarian assistance or disaster relief effort, which would be considering multiple sensors and unstructured reports from groups of people. This latter example has a greater role for people, social systems and their interactions. This is an evolving area, as the forms of human interaction are increasing, especially as new communication platforms appear. There has been some development into ontologies for CPSS modelling, although much of this is sourced from a few limited authors [8,58].

### 2.2. Social Media Complexity

Social media exemplifies the construct of a Cyber–Physical–Social System. Social systems, regardless of technological integration, are regarded as complex systems [31] (p. 29). Complex systems are entities that consist of “many interacting parts”, and exist on the “edge of chaos” [59] (p. 1) and [60]. Social media is inherently reliant on layers of complex systems, often driven by people, and is fed by these principles [61,62]. Terms such as “the Internet of Minds” and “Humans as Sensors” have also contributed to understanding social interaction with CPS through an IoT perspective [63,64]. Furthermore, Maier et al. [65] discussed the role of humans as “neurons in a hive mind” as part of Society 5.0, enabled by the token economy. Subsequently, there is a strong link between the concept of social media and its intersection with the field of CPSS.

Complex systems are characterized by several behaviors and features. These aid in distinguishing complex systems from both chaos and complicated systems. Complicated systems contain a multitude of parts that are defined by ordered and simple rules [66]. Complex systems exist at the intersection between chaos and complicated systems, with sub-units potentially including both chaotic and complicated dynamics. Complex adaptive systems are a more specialized case of complex systems, although sometimes the terms are used interchangeably. Complex adaptive systems dynamically evolve with and exhibit adaptation within changing environments, meaning that the system will not always react consistently to the same stimuli [67] (p. 2). They are distinct from the features of systems of equilibrium or homeostasis. Complex adaptive systems contain agents within their system boundaries, which are semi-autonomous units that seek to optimize based on a schema of prescriptive actions and rules [68] (p. 18) and [69] (p. 85). The behavior of these agents can subsequently display patterns at the system level, not all of which are predictable or repeatable. The degree of complexity experienced by a system can be measured by its difficulty in description, its difficulty in creation, and its degree of organization [70]. Many complex adaptive systems change over time, with changing optimized organization depending on the current context or environment. Many human and social interactions form parts of larger complex adaptive systems, with wider environment changes influencing both individual and system behavior [71].

The features of complex systems identified in the literature are varied; however, there are some key commonalities between interpretations [31,32,33,34]. The boundaries between these systems and their sub-units are sometimes blurred [34]. Complex systems often contain the features of self-organization; interdependence; hierarchy and scale; evolution, feedback and adaptive interaction; unpredictability and non-linearity; sensitivity to initial conditions; and emergence [31,32,33,34]. Social media is clearly established in the literature as a complex system, which creates additional challenges for the conceptualization and modelling of the system within the scope of CPSS [72] (p. 374). Social media case studies have exemplified how its emergence has had far-reaching consequences on political, economic, and social outcomes.

Conceptual frameworks and analysis of historic social and economic events fueled by complex social media interactions have been studied, including such diverse events as the Arab Spring, Euromaidan, and Gamestop [73,74,75,76,77]. Furthermore, the evolution of news consumption through social media has increased opportunities for the generation of ‘Fake News’. This has been a contributing factor to real-world phenomena including politically influenced ideological polarization within the social population [78,79,80]. CPS theory is an opportunity to examine emergence within social media, and therefore model the complexity in CPSS. There are further epistemological questions regarding complex CPSS, with challenges in identifying and understanding the inputs, processes, and outputs that social agents have within these systems. Subsequently, modelling and understanding social media as a form of complex CPSS is a field warranting further investigation. This study attempts to understand the distribution of research relating to this topic and determine where research gaps and opportunities exist to further its conceptual development.

## 3. Current State of the Art

This study was conducted via a mixed methods approach, with both quantitative and qualitative data analysis. It involved surveying search results from the Scopus database related to the research topic, statistical analysis of those results, exporting result metadata to file for topic cluster analysis in VOSViewer, and finally a conceptual analysis of key research papers identified. VOSViewer and similar visualization tools have been widely used in the preliminary analysis and identification research opportunities in several academic domains [81], including specific IoT environments [82] and computational physical chemistry [83]. There are also several other tools designed to perform similar meta-analysis of academic media via citations, keywords and themes. These include CitNetExplorer [84] and SciMAT [85]. These tools, first developed a decade ago, are seeing increased and significant use in publication analysis, in particular for environmental scans and surveys. Visualization has become an important tool across understanding research domains as varied as deep learning in autonomous vehicles [86], understanding how COVID-19 has shaped research [87], and the links with Augmented Reality in learning [88]. This work has chosen VOSViewer for use, but does not preclude the use of other tools for future comparative analysis. Through the methods discussed, the aforementioned research questions were analyzed and able to be answered.

### 3.1. Distribution of Current Research

Data collection occurred by searching for key terms within the Scopus database, and analysis of the results’ bibliographic data. The search phrases utilized are detailed in Table 1. The search query was applied to the article title, abstract and keywords. These terms were used because they related to answering the aforementioned research questions. They focus on understanding the distribution of research across these fields, whilst also highlighting the degree of intersection into the chosen research area.

### 3.2. Statistical Visualization of Current Research

**Figure 2:**
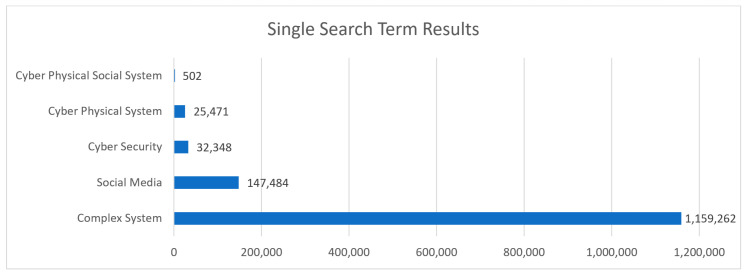
Single search term results.

From the Scopus data sets the degree of research within the chosen fields is quantified and visualized. Figure 2 illustrates the comparative size of each search result. The field of complex systems is the largest and most comprehensive. This implies how this field is endemic and underlies many other research areas. As a subset of CPS, CPSS predictably returned a smaller results size. In its early stage, CPSS is mainly focused on exploring potential applications across various domains, including smart cities, healthcare, transportation, and manufacturing. The CPSS trend focuses on studying how to merge traditional physical systems with cyber intelligence, communication capabilities, and social behavior analysis.

The intersection between CPS and CPSS involves exploring how technology and human behavior interact and influence each other, considering the security by design of their elements. For example, in a smart city scenario, CPS would include sensor networks and data processing systems to monitor and control infrastructure, while CPSS considers how people will interact with these systems, such as through social media platforms or mobile apps that provide information about the city’s status and encourage citizen engagement. Understanding the intersection between CPS and CPSS is important for designing more efficient and user-centric systems, as well as addressing ethical, legal, and societal implications. It involves studying complex socio-technical systems to ensure that technological advancements are aligned with human needs, values, and social structures.

**Figure 3:**
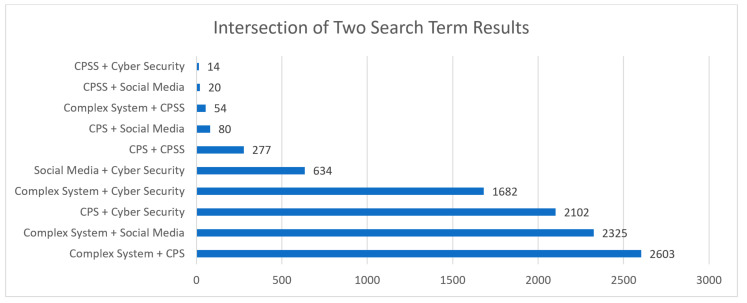
Dual term search results.

CPS are systems that combine physical components with computational elements to enable data sensing, processing, and control, with applications ranging from smart homes and energy grids to autonomous vehicles and industrial automation. CPSS, on the other hand, extends CPS by considering not only the technical aspects but also the social interactions, human behavior and cyber security within these systems. Figure 3 presents the intersection of the different fields of research. The most common fields of intersection were *complex systems and CPS*, followed by *complex systems and social media*. Conversely, intersections between CPSS and other topics were among the lowest statistics. These statistics align with the general trends identified in Figure 2, as topics that had a larger quantity of papers were more likely to have a larger intersection of multiple paper topics in Figure 3. With CPSS being a subset of CPS, the results aligned with this assumption that the subset topic’s intersection with other fields of research would be less than the overarching parents’ topic intersection.

Table 2 shows the distribution of papers with an intersection of multiple search topics. Of note, CPSS intersection with multiple other research fields yielded no results in any combination. This indicates that there is potentially a research gap in these areas, which is worth exploring further within the literature.

### 3.3. Network Analysis of Current Research

The bibliographic data from the Scopus database results were then assessed utilizing network analysis maps in the tool VOSViewer. They were analyzed utilizing the co-occurrence method using keywords of the papers. The counting method specified was ‘full counting’ and no thesaurus file was used. Phrases were prioritized for analysis by lowering the occurrence threshold until the number of occurrences was simultaneously minimized, whilst remaining above 30. For inputs that could not meet the threshold of 30, all terms were used. This meant that most maps had approximately 30 terms. The Scopus search terms for each result set were discounted from the final data set, and any outlying data that did not connect to the main graph was also discounted.

### 3.4. Conceptual Analysis of Most Influential Research

The conceptual analysis involved a review of the key topics utilizing the Scopus search outputs for the *CPSS* search term. The intent of this analysis was to determine novel research within the field and highlight the most influential papers. Selection criteria were applied to the results to narrow the focus of the review. The following terms and criteria were excluded from the analysis: survey papers, conference proceedings/journal introductions/opening chapters, references that did not contain abstracts, papers that did not include CPSS in their abstract, references whose full text could not be found, and papers that were not written in English. After this, the top 30 highly cited papers were selected for the development of a CPSS influence concept map.

This process was then repeated with the original *CPSS* Scopus search results data set. In this iteration, research papers were limited to the years 2021–2023 to assist in identifying the most novel and highly influential recent papers. References were chosen that had 5 or more citations for review, with the final reference count for analysis being 25.

### 3.5. Analysis of Current Research

The distribution of the research topics was significantly varied. Fields such as *complex systems* returned over a million results, whereas newer terms such as *CPSS* returned less than one thousand. CPSS was also highlighted as a significantly small subset against the wider field of CPS, with approximately 500 papers compared to 25,000 in the search results. These results indicated that the field of CPSS is relatively small compared to other established related research areas, and may warrant further expansion and investigation.

When considering the intersection of multiple search terms, as highlighted in Table 2, there were limited multi-disciplinary papers that addressed the research topics defined in Table 1. In particular, the queries that searched for the intersection of CPSS with multiple other research topics all produced zero search results, indicating that there is a significant research gap at the overlap of these key areas. As an emerging research area, this indicates that applications and research into CPSS require increased focus and commitment in order to understand the interrelation of complex CPS in the case of social media implications for cyber security.

## 4. Recent Technologies with CPSS

Recent technologies, including big data, IoT, machine learning and social media platforms have been employed in conjunction with CPSS. These technologies with CPSS, have revolutionised various fields by allowing advanced data analysis and decision-making processes. Big data allow massive volumes of structured and unstructured data that cannot be effectively processed by traditional methods. With recent technological advancements, it is now possible to collect, store, and analyze these vast amounts of data. Machine learning (ML), a subset of artificial intelligence, is a methodology that allows computer systems to automatically learn and improve from experience without being explicitly programmed. By utilizing ML algorithms, they can make predictions, classify data, and discover patterns within big data sets. Social media plays a significant role in the collection and generation of big data. Platforms like Facebook, Twitter, and Instagram provide vast amounts of data generated by users worldwide. These data can be utilized to study human behavior, sentiment analysis, and societal trends.

**Figure 4:**
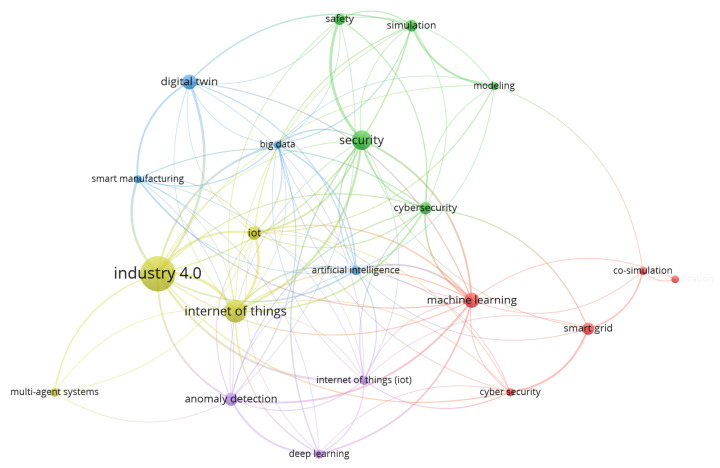
Complex system + Cyber–Physical System clusters (2019–2020).

The integration of these technologies allows monitoring and control of various systems, ranging from smart cities, transportation networks, and healthcare systems, to environmental monitoring. Figure 4, which is the *Complex system and CPS* intersection map, exemplifies this trend. Every cluster map generated throughout the analysis, with the exception of the *CPS and CPSS* intersection map and the *CPSS and social media* intersection map, includes at least one of the aforementioned topics. This indicates a significant research focus across the literature on these key topics.

**Figure 5:**
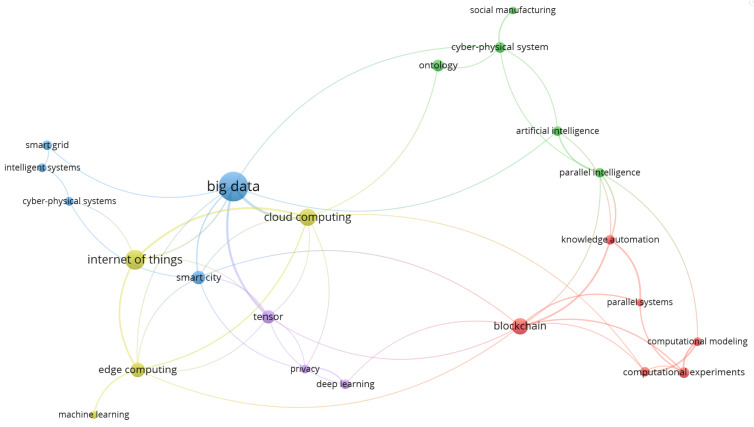
CPSS research clusters (2018–2021).

When drilling down into the research topic of CPSS, the largest areas of research focused on big data and the IoT; see Figure 5. In this model, the oldest research areas included IoT, intelligent systems, and social manufacturing. The newest areas included computational modelling, the Metaverse, and computational experiments.

**Figure 6:**
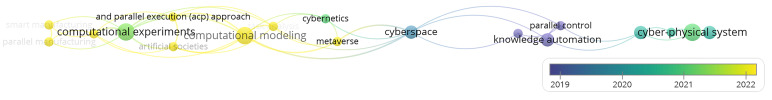
CPSS + complex systems time overlay (2019–2022).

When considering the intersection of topics, research that focused on both *CPSS and complex systems* was clustered across a linear scale. The oldest research topics were located in the center, with newer research extending to either side, as shown in Figure 6. The oldest research was associated with knowledge automation and parallel control, with newer research associated with computational modeling, smart manufacturing, task analysis and the Metaverse.

## 5. Most Influential Research Focuses

**Figure 7:**
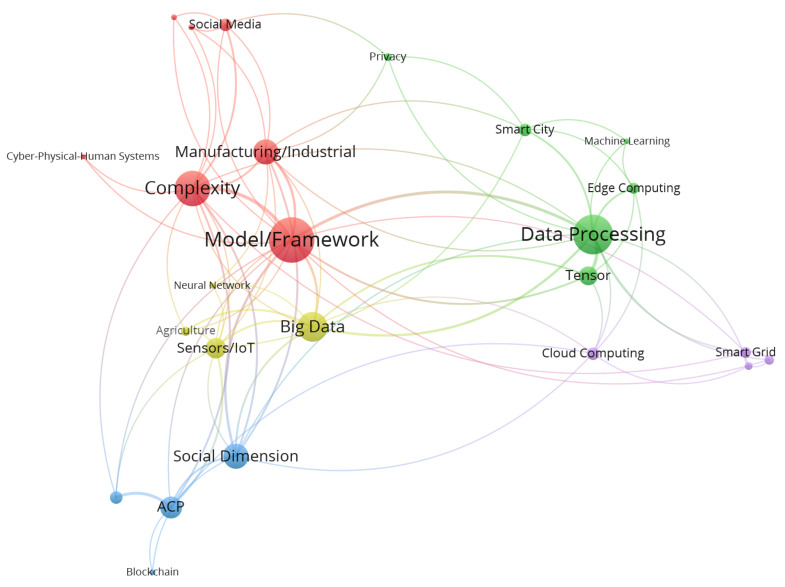
Most influential research clusters in the field of CPSS.

Within the field of Cyber–Physical–Social Systems, there are several areas of research focus apparent within the literature. Figure 7 highlights the top thirty influential CPSS papers, based on citations, and their topic clusters. Modelling CPSS through frameworks, simulations and data models is a key research area within the most influential CPSS papers. The complexity considerations of CPSS are identified and discussed throughout the literature. There was also a significant research focus on data processing implementations and optimization in areas such as resource allocation and capacity management.

The most influential case studies within the field related to manufacturing and industrial applications, smart cities, social media, and the smart grid. The most influential enabling technologies identified included tensor, edge computing, the IoT, and artificial societies, computational experiments, and parallel execution (ACP).

## 6. Role of CPSS with Recent Smart Applications

The concept of CPSS was noted in the literature to be linked regularly to several smart applications such as smart cities, smart healthcare, blockchain technologies, etc. The most regularly referenced included industrial applications and smart city implementations. These included manufacturing and Industry 4.0, smart cities, and blockchain tokenization [89,90,91,92]. Smart city implementations are also referred to in the highest CPSS paper citations [15,18,92,93]. Other CPSS implementations explored within the recent novel literature include smart wearable devices, data center cooling systems, industrial chart data processing, and electricity savings [22,94,95,96]. The gaming industry is additionally identified as a CPSS, with research in this space aligning with opportunities to crowd-source task allocation and optimization problems relying on the interaction of people with digital systems [97]. Future aims of this work include crowd-sensing via real user testing in order to generate incentive strategy evaluations [97].

Industrial implementations are identified in the literature as novel case studies for CPSS within the wider context of Industry 4.0. Performance monitoring is one area of development, where a CPSS framework is complimented to enable greater fidelity to detection and monitoring processes [90]. Evolving out of Industry 4.0, Industry 5.0 is an additional paradigm through which the concept of CPSS is discussed, integrating with other enabling technologies such as 6G, tokenization, robotics, tactile internet and collective intelligence [65,91]. These novel ideas also further link to the concept of Society 5.0, where CPSS’s aim becomes to enhance wider system harmony [65,91]. However, Maier’s work is challenged by the semantics of CPSS, sometimes identifying human-in-the-loop centric systems, which in some scenarios may align better with categorization as Cyber–Physical–Human Systems (CPHS) [91]. This cross-association indicates there is potentially a need for further work in defining the ontologies that separate these two sub-types of CPS, and the factors that conclusively assign a system to one definition over another.

A portion of the research related to industrial applications of CPSS appears to be more closely aligned with a traditional CPS or Industrial CPS (ICPS), instead of the social dimensions and metrics traditionally included within the CPSS construct. In some papers, this manifested in a Cyber–Physical–Human System (CPHS) context, where people’s interactions with the industrial system were considered, but frameworks did not include metrics or sensors relating to complex human social systems, and instead focused on predictable defined human-in-the-loop switch case interactions [9].

### 6.1. Data Processing and Resource Allocation

Innovations and novel implementations of CPSS in the recent literature often focused on improving resource allocation or optimizing data mining within large data sets as enabled by sensors and IoT. CPSS is identified as having significant data processing and analysis considerations due to its heterogeneity, growth, and dimensionality [98].

Collaboration is one research area that aims to improve CPSS data processing in order to support multi-goal optimization solutions [98]. Furthermore, Wang et al. introduced and tests the performance of algorithms in their role of processing CPSS big data in smart cities, including singular value decomposition (HOSVD), ring-based tree algorithms and tree-based tree algorithms [92]. This research identifies the need for future computational efficiency studies to improve the algorithmic processing of CPSS data in wide-scale applications [92].

Machine learning is a further tool used to build solutions to big data for CPSS processing challenges, with algorithms leveraging computing constructs such as cloud computing, edge computing, and distributed training in order to enable systematic processing [92]. Linear Regression is another machine learning technique that is applied to data analysis for CPSS, with application implications to the cyber security of data processed in untrustworthy environments, such as cloud servers or the blockchain [99]. Whilst privacy and security concerns were identified as a focus point for experimentation in CPSS data processing, the authors noted that future work in this domain would need to continue to improve data privacy, verifiability of results, and fairness [99] (p. 3966). Parallel execution is used as one way to validate AI-processed CPSS data, with future work in this space highlighting the need for real-world accurate simulation systems, and validation of outputs against read scene data [93]. Notably, the recent novel literature regarding CPSS data algorithms does not address newly emerging fields such as federated learning, where collaborative machine learning occurs across multiple decentralized clients utilizing architecture such as IoT [100,101]. The discussions regarding applications of such algorithms are limited, and present a further opportunity for research growth and maturity [96].

The processing of data to feed recommendation services as part of the CPSS construct is also considered in the literature, with algorithms built to process and analyze user characteristic data in diverse scenarios [51]. Future work in this area focuses on testing existing solutions with larger and more complex data sets to ensure quality of service in wide-scale applications [51] (p. 3858). Diverse scenario processing in the context of CPSS is an emerging space. Through the conceptual analysis, there were limited novel studies identified that challenge legacy machine learning algorithms with alternatives such as federated learning.

Data for CPSS processing must also be considered with a system-of-systems mindset, where multiple subsystems with varying data sets must be interconnected and processed. Within the smart city case study, this problem set is exemplified, with sub-systems including power grids, human social systems, and SCADA systems [102]. Hazard identification for safety is one research goal of these studies, where risk analytics are derived from interdependent network theory. However, future work is required in the fields of system resiliency in the case of cyber attacks in order to provide accurate hazard assessments [102] (p. 15).

Tensors are identified as one avenue for enabling heterogeneous data learning and optimization for CPSS [103,104,105]. Considerations for how to optimize the modelling of CPSS that utilize Intelligent Edge Services (IES) as part of an edge computing construct experience barriers in the form of security-efficiency compromises, energy consumption effects, and cost [104]. Future work in this optimization space needs to consider the secure optimization of multiple objectives, due to the complexity of CPSS, in order for CPSS-related price execution to become more economically viable [104] (p. 44). Additionally, the application of leading-edge machine learning technologies, such as federated learning, is a further opportunity for CPSS data processing research, especially in consideration of the joint foundational applicability of IoT to the two fields [101,106].

Algorithms that leverage sensor technology are identified in the literature to demonstrate more accurate classification results compared to legacy models; however, they highlight challenges to the flexibility of such models when training data constraints are introduced [105]. This is of particular concern when considered within the CPSS construct, where emergence may introduce unpredictable results to algorithm outputs, with the potential introduction of new data sets that do not nearly align with trained parameters.

The social component of human-generated information is also a data processing challenge, with sensors such as social media posts requiring analysis and comprehension in the digital space. The use of models such as Natural Language Processing needs to evolve to meet this need, as data sets may exhibit emergent or unpredictable behavior [63]. Opportunities within this sphere are posed through solutions such as Zero-Shot algorithms, that can potentially analyze data from unseen classes; however, continued development into the processing of unlabeled data is required to improve performance, especially in relation to semantics and knowledge graphs [63].

### 6.2. Cyber Security Implications

Cyber security and privacy concerns relating to data in IoT-enabled CPSS are of note within the literature and are explored through multiple case studies and implementations. Integration of social and IoT technologies into CPSS presents an opportunity to develop frameworks that conceptualize Enterprise Digital Transformation, such as seen in Mendhurwar and Mishra’s framework [107]. Their model specifically identifies not only the inter-dependencies between social agents and IoT sensors as part of CPSS but highlights the cyber security challenges associated with this digital transformation process [107]. They further identify that future work in this space may consider case studies that can provide insight into the nuances of specific applications and deliverables within the industry, considering factors such as entity needs and security posture [107].

Data analysis in industrial applications is also at the leading edge of research regarding CPSS, especially in conjunction with privacy considerations associated with data mining [89]. Algorithms such as the high-order Bi-Lanczos (HOBI-Lanczos) approach demonstrate an ability to securitize data and protect user privacy. Future work in this space includes exploration of energy implementations and collusion approach considerations [89]. Gati et al. [41] explored this concern regarding privacy preservation within CPSS, with their solution leveraging deep learning techniques in order to process private data. They propose a differential privacy framework that is applicable across multiple use cases such as smart agriculture, smart healthcare, and smart transportation [41]. Privacy protection and secure data considerations are also highlighted within CPSS research [96,103]. Human Activity Recognition (HAR) utilizing machine learning is one avenue for enabling secure processing of data framed through wearable devices [96]. Future research in this field involves investing in greater efficiency of algorithms, in addition to more personalized modelling whilst maintaining user privacy [96]. Additional challenges in this space involve the privacy preservation of data and processing, in relation to centralized models, and also the collection and integration of nonindependent and identically distributed (non-IID) data.

CPSS is also identified in the literature as an opportunity for attack vectors, which have the potential to compromise the cyber security of systems and their users. In these contexts, variations of existing attack vectors such as social engineering and malicious code execution are applied within the CPSS context, rather than a new attack class being defined. For example, Yang et al. propose the use of a neural network, called MCNN, to identify digitally manipulated ‘fake’ images in order to reduce the attack surface for anti-forensics tools [108]. Research such as this highlights the potential for CPSS to have real-world impacts; however, current works tend to focus on specific case studies rather than wider cross-domain conceptual applications. As people and technology integrate further in the Cyber–Physical–Social world, this poses additional opportunities for cyber attacks, as each edge between nodes in the CPSS presents additional potential attack vectors.

## 7. Research Challenges and Lessons Learned

As discussed above, the majority of novel contributions to the literature surrounding CPSS over the last three years can be categorized into one of three research fields. These areas of concern are broadly considered as case study CPSS implementations, such as smart cities; data processing considerations, such as efficiency algorithms; and cyber security implications, such as user data privacy concerns.

Through this critical survey and analysis of the literature, several significant research gaps and opportunities were identified and synthesized. Research gaps that specifically addressed the research questions included a lack of social metric inclusion in system-of-systems modelling for CPSS, a need for a CPSS ontology that includes consideration of the other intersecting research fields, and the inclusion of complex system dynamics within frameworks, including an understanding of emergent effects within the cyber, physical and social domains of CPSS. Additionally, CPSS research gaps were identified that related to comprehensive holistic case study analysis and cross-silo analysis of enabling emergent technologies including smart contracts and the blockchain, federated learning, sensor design and distribution. Furthermore, the existing research rarely addresses the impact of opaqueness within complex CPSS and how that may affect the accuracy and reliability of machine learning algorithms and decision support systems.

There is a lack of highly cited novel papers that specifically focus on developing frameworks or ontologies related to the social component of CPSS. Many papers that reference CPSS in their keywords or abstract often use this term to exemplify how a system can be considered a CPSS, without necessarily focusing on the nature of CPSS as part of their research. Multiple CPSS papers in the literature name the system in question as a CPSS, but then focus research on multidisciplinary fields such as algorithms for resource allocation or case studies such as smart grids [95]. Models and frameworks for CPSS as identified in the most recent novel literature also highlighted implementations focused on either parallel execution, or only considered the Cyber–Physical components of the system instead of including the human social metrics. Social data within the context of CPSS are focused upon little within the literature, with the outputs usually measured through methods such as social media posts or interactions with CPS system boundaries, such as interfacing with a smart city application.

There is, therefore, a research opportunity to aid in defining how these social data can be measured, and the subsequent challenges of processing such heterogeneous unlabeled data at scale. There is subsequently a need to identify how human metrics can be effectively modelled within larger CPSS frameworks, which include their social behaviors, attributes, and subsystems. Furthermore, there was evidence in the literature that CPSS was defined in broad and sometimes contradicting ways between different authors, with the comparison of CPHS to CPSS as an example of this. Analysis of the literature further highlighted some related research terms that could contribute to understanding the nature of CPSS. Terms such as persuasive computing, Society 5.0, Internet of Minds, and cybernetics also appeared to constitute part of CPSS as a concept, thus warranting further investigation and linking towards this larger CPSS ontology.

Whilst complexity is defined as an essential component of CPSS as per its originating conception, features of complexity such as emergence are rarely considered within the literature [10]. When machine learning is considered part of a CPSS application, the feature of emergence is conceptually at odds with the traditional design of training data, which tend to be predictable. Additionally, algorithm design is often considered within a data mining/processing context instead of from the context of human interaction and social media analytics. Finally, there is a lack of research focus associated with understanding multi-domain effects caused by the complexity of data within CPSS, particularly in the social space, especially when these effects are related to emergence. Social systems are inherently complex, albeit self-organizing, and subsequently CPSS research needs to consider the features of complexity within its models and frameworks. As an example, leveraging technologies such as machine learning and artificial intelligence to assist in modelling and data processing for CPSS is an opportunity. However, complications arise when considering the effects of emergence on pre-trained models, and unstructured, unlabeled human metric data sets.

There is a research need for future work that directly addresses these concerns relative to human metrics, an inclusive research ontology, and complexity considerations. This research would have to rely on the assumption that CPSS are inherently different to CPHS and that social systems are included within the CPSS boundary, instead of simply interfacing with it. The development of such a framework would then require testing against an established real-world CPSS that includes real social interactions for validation purposes, such as social media.

The process used to develop this work prioritized impact based on the number of citations individual publications have received. This approach therefore focuses on what has already been deemed impactful within the academic communities. Subsequently, there were some very recent publications that did not fall within the catchment of the analysis, but represent the leading edge of literature published within the last year. Such research still should be considered in future work in the field of CPSS.

Additionally, some of the existing novel literature focuses on fields relating to this paper’s research question. For example, Che et al.’s work into tensor factorization for fake news detection does not explicitly identify the system in question as a CPSS, but still offers valuable insight into CPSS research [109]. Other recent non-CPSS explicit literature that may contribute to CPSS future research related to this paper’s research focus include studies pertaining to modeling misinformation and bias within social networks and those that address emergence within CPS constructs [110,111,112,113,114,115]. Additionally, emergence in the field of cyber security is an identified area of research interest that is worth exploring within the context of CPSS and the IoT.

Ultimately, the field of Cyber–Physical–Social Systems is growing, which has the potential to underpin a multitude of cross-domain applications from healthcare to politics to algorithm optimization to Industry 4.0. Thus, building effective frameworks to understand this field and its cyber security implications is fundamental to ongoing reliance in both the cyber, physical and social spheres of influence.

## 8. Conclusions

The research field of CPSS is in an evolving state. Definitions, applications, and understandings of the concept are fluid and sometimes contradicting. This is exacerbated by its cross-applicability to multi-disciplinary applications, enabling silos of ontological understanding. However, the research in the field clearly points towards constructs of CPSS being a key enabler for future technological development and implementation into digitally enabled society, and thus warrants continued research and synthesis.

This work has shown that there is are several areas of CPSS that require additional research in order to reach its potential. This work has noted that the community is yet to standardize on common terms and definitions. This is not unexpected with such an emerging area. The development of structured representation mechanisms, such as formal ontologies, are one area of potential future research, that would assist in alleviating some of these issues.

Similarly, the broad nature of CPSS requires both a balance of the holistic in addition to detailed perspectives. This balance is difficult to achieve. The nature of complex systems makes this difficult, and there is more work to be carried out to understand some of the interactions of and between different domains.

Finally, this work has outlined the need for additional cybersecurity research both across both each domain of interest and at the areas that these domains integrate. The social aspect of cybersecurity is well known, but as CPSS evolves, so too will the challenges and opportunities in this area.

Social factors can heavily influence global current affairs, and thus the relationship between social agents and their Cyber–Physical counterparts presents a potentially volatile vector for influence, feedback, and emergence. Fake news, political events like the Arab Spring and Euromaidan, and economic events such as Gamestop all exemplify how Cyber–Physical Systems can have social components that affect outcomes in the real, physical world. Understanding how Cyber–Physical–Social Systems exist and operate is imperative to predict the emergence and the subsequent cyber security implications of these events.

## Figures and Tables

**Table 1:** Scopus search phrases.

Report Term	Scopus Search Phrase
Complex system	“*Complex system*” OR “*Complexity*”
Cyber–Physical System/CPS	“*Cyber Physical System*” OR “*Cyber–Physical System*”
Cyber–Physical–Social System/CPSS	“*Cyber Physical Social System*” OR “*Cyber–Physical Social System*”
Social Media	“*Social media*”
Influence	“*Influence*”
Cyber Security	“*Cybersecurity*” OR “*Cyber security*”

**Table 2:** Multiple term search results.

		CPSS	CPS
Three Terms	Complex system + Social Media	0	3
Three Terms	Complex system + Cyber Security	0	182
Four Terms	Complex system + Social Media + Cyber Security	0	0

## Data Availability

No new data were created or analyzed in this study. Data sharing is not applicable to this article.

## Abbreviations

The following abbreviations are used in this manuscript:

MDPI	Multidisciplinary Digital Publishing Institute
DOAJ	Directory of open access journals
TLA	Three letter acronym
LD	Linear dichroism
IoT	Internet of Things
CPS	Cyber–Physical System
CPSS	Cyber–Physical–Social System
C2	Command and Control
CPHS	Cyber–Physical–Human System
HOBI-Lanczos	High-Order Bi-Lanczos
IES	Intelligent Edge Services
HAR	Human Activity Recognition
Non-IID	Nonindependent and Identically Distributed
ICPS	Industrial Cyber–Physical Systems
ACP	Artificial Societies, Computational Experiments, and Parallel Execution
